# Critical Care of the Adult With Congenital Heart Disease

**DOI:** 10.1016/j.jacadv.2025.102081

**Published:** 2025-08-27

**Authors:** Christopher W. Valle, Amanda C. Garfinkel, Jonathan Buber, Anitra W. Romfh, Andrea M. Elliott, Jonathan N. Menachem, Jennifer Nelson, Peter C. Laussen, Jane Heggie, Cameron Dezfulian, David Morrow, Anne Marie Valente, American College of Cardiology Critical Care Cardiology Section and Adult Congenital and Pediatric Cardiology Section

**Affiliations:** a Department of Cardiology, Boston Children's Hospital, Boston, Massachusetts, USA; b Division of Cardiovascular Medicine, Department of Medicine, Brigham and Women's Hospital, Boston, Massachusetts, USA; c Harvard Medical School, Boston, Massachusetts, USA; d University of Washington Medical Center Department of Medicine, Division of Cardiology, University of Washington School of Medicine, Seattle, Washington, USA; e Department of Pediatrics, Pediatric Cardiology, Stanford University, Palo Alto, California, USA; f Lucile Packard Children's Hospital, Division of Cardiovascular Medicine, Department of Medicine, Stanford Health Care, Stanford University, Palo Alto, California, USA; g Section of Critical Care Cardiology, Cardiovascular Division, University of Minnesota, Minneapolis, Minnesota, USA; h Division of Cardiology, Vanderbilt University Medical Center, Nashville, Tennessee, USA; i Department of Cardiovascular Services, Nemours Children’s Hospital, Orlando, Florida, USA; j Department of Anesthesiology, Critical Care and Pain Medicine, Boston Children's Hospital, Boston, Massachusetts, USA; k Department of Anesthesia and Pain Medicine, Peter Munk Cardiac Centre, Toronto General Hospital, Toronto, Ontario, Canada; l Department of Pediatrics, Division of Critical Care, Baylor College of Medicine, Houston, Texas, USA; m Department of Anesthesiology and Critical Care, Baylor College of Medicine, Houston, Texas, USA; n Levine Cardiac Intensive Care Unit, TIMI Study Group, Cardiovascular Division, Department of Medicine, Brigham and Women's Hospital and Harvard Medical School, Boston, Massachusetts, USA

**Keywords:** ACHD, congenital heart disease, critical care, Fontan, surgical outcomes

## Abstract

Advances in the treatment of congenital heart disease (CHD) have led to dramatic improvements in survival for individuals with CHD. While adults with CHD represent a small percentage of admissions to the intensive care unit (ICU), the critical care needs of this population will grow as this population ages and develops increasingly complex cardiac and noncardiac conditions. Adults with CHD require special care in the ICU because of both their unique cardiovascular conditions and the multi-organ dysfunction that often accompanies their cardiac pathophysiology. This review aims to summarize the current epidemiology of critical care for adults with CHD, describe key physiologic and management considerations in caring for adults with highly complex CHD (eg, Fontan circulation, systemic right ventricle, and Eisenmenger syndrome), identify cardiac and noncardiac risk factors for adverse outcomes following admission to the ICU, and define key research and educational priorities for the future care of this vulnerable population.

## Highlights

• The population of adults with CHD requiring critical care is growing in total volume and complexity.
• Adults with CHD have unique hemodynamic and physiologic profiles with sparse evidence to guide critical care.
• Future research, training, and workforce development are needed to improve the care of critically ill adults with CHD.

Advances in the care of individuals with congenital heart disease (CHD) have led to improved survival to adulthood. While the incidence of CHD remains unchanged over decades, the prevalence of adults with CHD has grown dramatically and is expected to rise through 2050.^1^, ^2^, ^3^ Many adults with CHD live with subclinical organ dysfunction related to effects of their altered physiology and are vulnerable to the development of multiple cardiac and noncardiac conditions requiring admission to an intensive care unit (ICU).^4^ Given these patients’ medical complexity, they benefit from collaborative care by providers with adult CHD (ACHD)-specific training or in Adult Congenital Heart Association (ACHA) accredited centers.^5^ As the ACHD population continues to grow and as critical care cardiology emerges as a unique subspecialty,^6^ there is a need and an opportunity to develop care delivery systems, focused research priorities, and educational pathways for adults with CHD requiring critical care (Central Illustration).

**Central Illustration:**
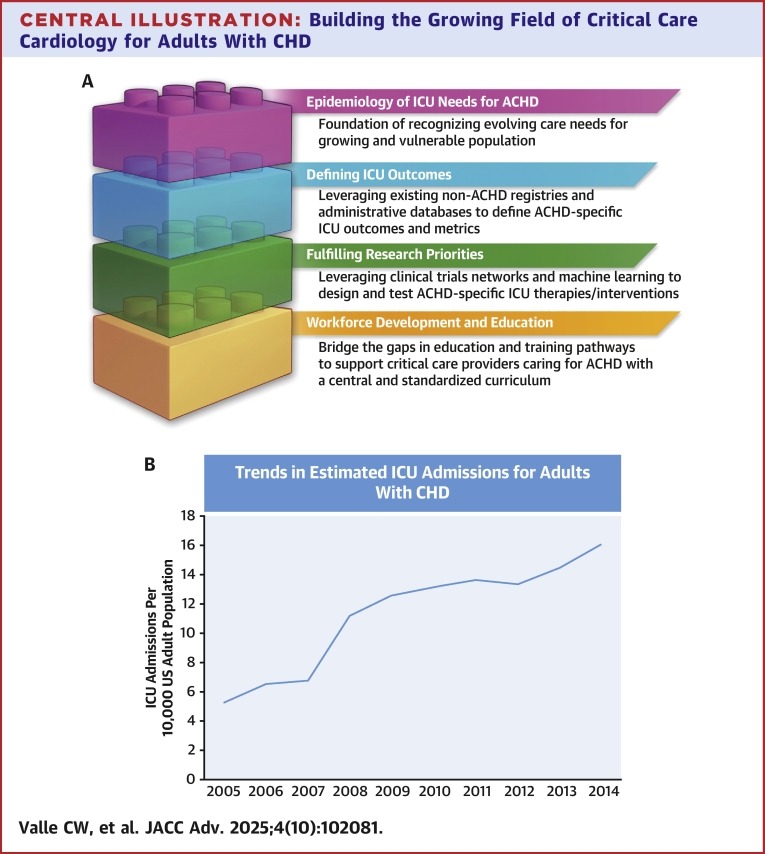
**Building the Growing Field of Critical Care Cardiology for Adults With CHD** (A) Building blocks of the field of critical care cardiology for adults with congenital heart disease (CHD) population. (B) Growing incidence of intensive care unit (ICU) admission for adults with CHD. Abbreviation as in Figure 3.

In this state-of-the-art review, we discuss the current epidemiology of ACHD ICU care, review core physiology and management pearls unique to the critical care of this population, highlight available data on outcomes, identify key gaps in evidence, and address considerations for workforce development and education to meet the needs of adults with CHD in the ICU.

## Epidemiology of care of adults with CHD in the ICU

The epidemiology of the critical care of adults with CHD can be broken down into basic questions: Why, Who, When, and Where? For the purposes of this state-of-the-art review, we aim to describe critical care administered for both acute medical illness—typically provided in the adult medical ICU (MICU), adult cardiac care unit, or pediatric cardiac ICU (CICU)—as well as critical care provided following surgical interventions—administered in the cardiac surgical ICU (CSICU) or surgical ICU (SICU).

### Why are adults with CHD admitted to the ICU?

Compared to adults with acquired heart disease, adults with CHD are significantly more likely to be admitted to the ICU for management of heart failure, atrial arrhythmias, noncardiac problems including sepsis and acute respiratory distress syndrome, and pulmonary hypertension.^7^, ^8^, ^9^ This difference in primary admission diagnoses is generally driven by younger age and a unique profile of noncardiac comorbidities, including higher rates of pulmonary hypertension and liver disease.^10^ Additionally, there is a high burden of bacterial endocarditis in this population, upward of 22 to 47 times that of adults with acquired heart disease, owing to a large proportion of patients having implanted prosthetic material (eg, conduits, valves, electrophysiologic devices).^11^ Many of these patients require medical critical care in the setting of acute sepsis and cardiogenic shock as well as surgical critical care following surgical replacement of infected valves and prosthetic material.^12^

### Who are the adults with CHD admitted to the ICU?

A population-based study from the province of Quebec found that over a 5-year period, 16% of adults with CHD were admitted to an ICU, with a median length of stay of 5 days,^13^ with rates of ICU admission being more than two-fold higher for severe ACHD compared to less complex forms of CHD. A similar total number of days in the ICU was reported in a study of ∼12,000 adults with CHD in Israel.^14^

While these studies do not report the proportion of patients admitted following surgical operations vs those for unplanned medical diagnoses, there are epidemiological studies that describe the surgical ICU needs of this population. Evidence from the Netherlands revealed that 1 in 5 ACHD patients, median age 33 years, required surgery over a 15-year period.^15^ A follow-up analysis from the same database suggested that upward of 43% of adults with CHD will require surgery by age 60 years.^16^ In the United States alone, recent analyses of the Society for Thoracic Surgeons database identified over 150,000 surgical operations for adults with CHD over 10 years.^17^^,^^18^ As nearly all cardiac surgical operations in this population will require ICU admission postoperatively, these studies point to an expanding need for surgical ICU capacity for adults with CHD.

### When are adults with CHD admitted to the ICU?

Adults with CHD may be admitted to the SICU and CSICU throughout their lifespan for acute episodes of surgical critical care. In contrast, admissions to the CICU for management of heart failure, arrhythmia, or pulmonary hypertension may punctuate the lifespan but can also be a harbinger of the end of life. Adults with CHD are likely to be admitted to the ICU in their final months to years of life. For example, ICU admission at a quaternary center approached 40% in the final 30 days of life, a rate significantly higher than that for adults with terminal cancer or heart failure secondary to noncongenital causes.^19^ Investigators from Belgium found similarly high rates of ICU utilization at the end of life in adults with CHD, 19% of whom required ICU admission in the final year and 15% in the final 30 days of life.^20^ These studies highlight the need to develop a cohort of clinicians skilled in palliative care to help this vulnerable population navigate the final days of life with dignity.

### Where are adults with CHD admitted to the ICU?

**Figure 1:**
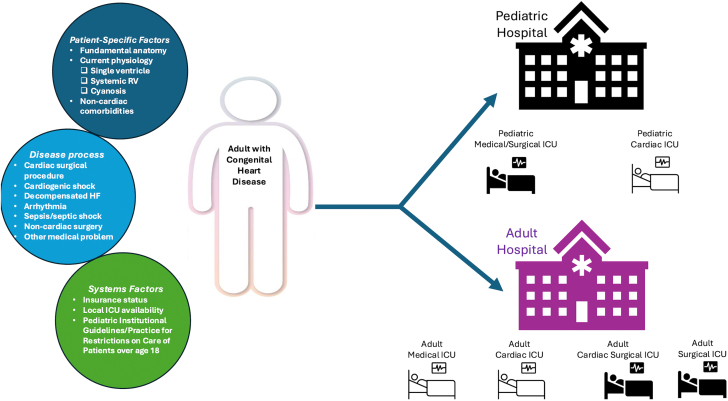
**Optimizing Site of Care for Adults With Congenital Heart Disease** Patient-level, disease-related, and systems factors determine site of care for adults with congenital heart disease (CHD) admitted to the intensive care unit (ICU). HF = heart failure; ICU = intensive care unit; RV = right ventricle.

Critical care management of adults with CHD may occur in various hospital settings. Depending on patient-level factors such as age, comorbidities, and complexity of diagnosis; systems factors related to insurance type and availability of ICU services in each geographic locale; and presenting disease-specific factors, adults with CHD can be admitted to the pediatric MICU, pediatric CICU, adult CICU, adult SICU, adult MICU, adult CSICU, or adult neurologic ICU (Figure 1).

To date, there are limited data to describe the factors that determine the optimal site of care for an individual patient. A single survey-based study describing current practices across 48 centers in the United States revealed that 56% of centers had access to expert consultation from an ACHA-accredited ACHD program.^21^ For nonsurgical patients requiring ICU-level care, 72% were cared for in adult CICUs. Among adults with CHD undergoing cardiac surgery, 72% received care in adult CSICUs. The presence of an ACHA-accredited program was associated with the availability of durable mechanical circulatory support (MCS), extracorporeal membrane oxygenation, and heart transplantation.

## Unique physiologic concepts in the critical care of adults with CHD

**Figure 2:**
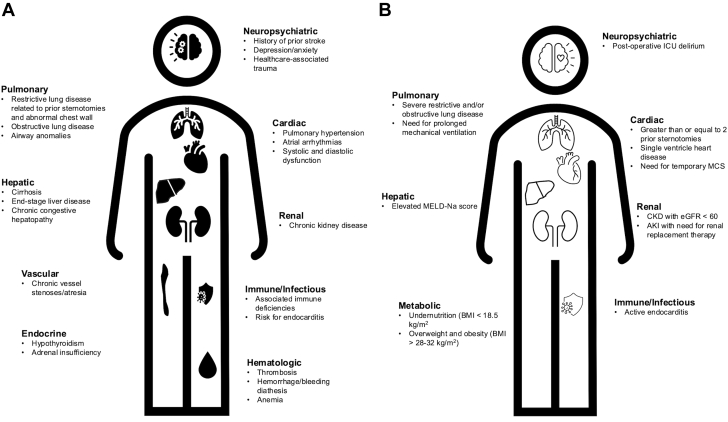
**Determinants of Intensive Care Unit Morbidity and Mortality** Noncardiac comorbidities impact management of adults with congenital heart disease (CHD) admitted to the ICU for medical indications (A). Risk factors associated with worsened postoperative morbidity and mortality in adults with CHD undergoing cardiac surgery (B). AKI = acute kidney injury; BMI = body mass index; CKD = chronic kidney disease; eGFR = estimated glomerular filtration rate; MCS = mechanical circulatory support; MELD-Na = Model for End-Stage Liver Disease–Sodium score.

There is immense heterogeneity in CHD, ranging from simple shunt-based lesions to very complex univentricular heart lesions that were either not repaired or palliated to a Fontan circulation. For adults with CHD, their care needs in the ICU are driven primarily by the interplay of their presenting pathology with their underlying cardiovascular anatomy and prior interventions, often with significant influence of their comorbid end-organ dysfunction (Figure 2). While most intensivists cannot be expected to recognize and understand all forms of CHD, there are certain physiologic derangements for specific cohorts that arise frequently and demand a unique therapeutic plan.

### Caring for adults with Fontan circulation

The growing population of adults with Fontan circulation may present to the ICU with multi-organ derangement and unique hemodynamic profiles. Adults with Fontan circulation have a higher incidence of hospitalization and death due to heart failure compared to adults with other CHD as well as adults with acquired heart disease, often requiring hospitalization at a much younger age.^22^

In these patients, venous return drains directly via the inferior and superior vena cavae into the pulmonary arterial circulation due to the absence of a subpulmonary cardiac pump. After returning to the heart, cardiac output is supplied by a single ventricle, which must overcome both the systemic vascular resistance (SVR), as well as the pulmonary vascular resistance (PVR).^23^ As such, pulmonary vasodilator therapy can be considered in select acutely ill Fontan patients in the ICU, particularly those with evidence of elevated PVR or hypoxemia, but patient selection is critical and evidence remains limited. Moreover, long-term exposure to elevated central venous and ventricular end-diastolic pressures predisposes to hepatic congestion with attendant Fontan-associated liver disease and the potential for chronically diminished systemic vascular tone.^24^^,^^25^ In this setting, many patients stand to benefit in acute, critical illness from a vasopressor strategy that mitigates low SVR with less effect on the PVR (eg, vasopressin and norepinephrine) as opposed to one that favors inotropic support (eg, epinephrine and dopamine).

**Figure 3:**
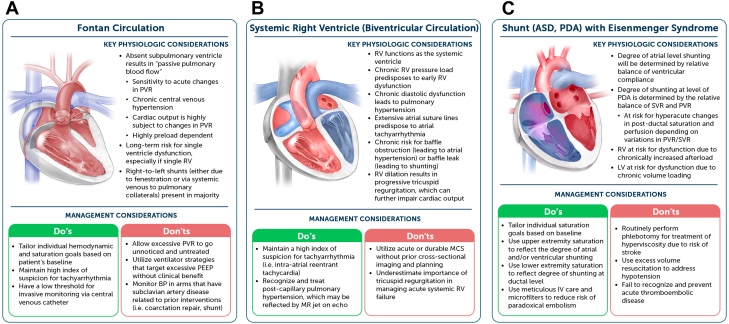
**Practical Pearls for the Critical Care of Complex ACHD** Physiologic considerations and management pearls are provided for adults with Fontan circulation (A), systemic right ventricle (B), and residual shunt resulting in Eisenmenger syndrome (C). ACHD = adult congenital heart disease; ASD = atrial septal defect; BP = blood pressure; LV = left ventricle; PDA = persistent ductus arteriosus; PEEP = positive end-expiratory pressure; PVR = pulmonary vascular resistance; SVR = systemic vascular resistance; other abbreviation as in Figure 2.

Within this physiologic construct, adults with Fontan circulation who are admitted to the ICU, especially those with cardiogenic shock and/or acute pulmonary pathology, must maintain a delicate balance of SVR and PVR, each of which may need to be managed independently (Figure 3A). For this reason, relatively routine ICU maneuvers like intubation and mechanical ventilation can be of much greater detriment to overall cardiac output than would be the case in adults with acquired heart disease. Intensivists must approach induction with caution, use intravenous fluids judiciously to ensure adequate preload, select anesthetic agents that achieve the desired effect without reducing vascular tone excessively, and be prepared to respond with inotropic, systemic vasopressor, and pulmonary vasodilator agents for prompt resuscitation as appropriate. Following induction and initiation of positive pressure, providers must also approach mechanical ventilation thoughtfully, avoiding excess positive end-expiratory pressure that could further impair venous return and lead to circulatory failure in this unique physiology.^26^ Moreover, providers should recognize the potential limitations of MCS in these patients. For example, peripheral extracorporeal membrane oxygenation may not drain the venous return adequately in patients with aortopulmonary collateral burden, preventing adequate ventricular decompression. Also, there is lack of evidence for use of other forms of acute MCS (eg, intra-aortic balloon pump, microaxial flow device) in Fontan circulation.^27^ Intensivists must also determine goal hemodynamics and oxygen saturations for these patients with single ventricle physiology, which often differ substantially from the general population.

Separate from the complex cardiorespiratory interactions, adults with Fontan circulation admitted with critical illness are at risk of noncardiac events related to subclinical organ dysfunction. Patients with Fontan circulation, especially those who have long-standing cyanosis, are predisposed to chronic kidney disease.^28^ They are additionally at risk of thromboembolic disease despite often having thrombocytopenia, which is typically mild. Venous thromboembolism can result in: 1) pulmonary embolism leading to impaired venous return and circulatory collapse; or 2) cerebrovascular accident in the case of paradoxical embolism via right-to-left shunting.^29^ For this reason, a large proportion of this population are receiving chronic anticoagulation and/or antiplatelet therapy,^30^ which may further complicate an acute episode of critical care.

### Caring for adults with systemic right ventricles

Distinct from single ventricle heart disease, the cohort of adults with D-transposition of the great arteries who have undergone a prior atrial switch (ie, Mustard or Senning operation) have a systemic right ventricle (RV) and are entering their fourth through sixth decades of life with attendant increase in complications of severe chronic heart failure and arrhythmias.^31^ This population is predisposed to the early development of heart failure as the morphologic RV develops fibrosis, dilation, tricuspid regurgitation, and systolic dysfunction with prolonged exposure to the relatively higher afterload of the SVR. They are additionally at risk for atrial arrhythmias and baffle obstruction. There is a distinct but related population of adults with “congenitally corrected” or “physiologically corrected” transposition of the great arteries who also have a systemic RV but carry a different hemodynamic profile related to high rates of associated defects and atrioventricular block.^32^^,^^33^ In the ICU setting, these patients merit special consideration given the potential limited efficacy for medical therapies for end-stage heart failure.^34^ In the absence of meaningful evidence to suggest otherwise, intensivists should approach the use of inotropic and vasoactive medications as they would for a patient with systemic left ventricular failure resulting in cardiogenic shock. Moreover, providers should recognize that while patients with acute systemic right ventricular failure are increasingly supported with acute and durable MCS (eg, microaxial flow pump or durable ventricular assist device; Figure 3B), there are unique anatomic features of the RV cavity that can complicate implantation leading to inadequate inflow/support, suction events, arrhythmias, and hemolysis.^35^

### Caring for adults with Eisenmenger physiology

Adults with Eisenmenger syndrome comprise up to 5% of ACHD cohorts in large tertiary centers.^36^ These patients have chronic cyanosis related to right-to-left shunting in the setting of pulmonary arterial hypertension. As such, ICU teams must target appropriate oxygen saturations relative to their baseline chronic hypoxemia. Moreover, acute presentations that require ICU care related to conditions that lower SVR (eg, sepsis) or raise PVR (eg, pneumonia, pulmonary embolism) can be life-threatening and difficult to rescue, by creating a spiral of progressive hypoxia which leads to increased PVR and further exacerbates shunting. Moreover, traditional salvage maneuvers for refractory hypoxemia in the noncongenital population could prove catastrophic if used in the wrong clinical setting. For example, the use of invasive mechanical ventilation with a high-positive end-expiratory pressure strategy can increase PVR if the lung alveoli are overdistended, and administration of sedation/paralysis can lower SVR through reduction in vascular smooth muscle tone.^37^ Each of these therapies might shift the balance between PVR and SVR leading to the negative feedback loop of further hypoxia and resultant adverse shunting. Thus, these patients benefit from thoughtful use of therapies to modify the PVR, such as pulmonary vasodilators, and SVR, such as systemic vasoconstrictors, while treating the primary condition driving ICU admission and tolerating a degree of acute on chronic cyanosis (Figure 3C). Additionally, providers must make highly individualized decisions regarding anticoagulation for each patient given the fine balance between clinical bleeding, especially from hemoptysis, and thromboembolic disease, as this population is at higher risk of paradoxical embolism and stroke in the setting of bidirectional shunting.

## Outcomes for adults with CHD in the ICU

### Medical and noncardiac surgical ICU admissions

Adults with CHD have higher morbidity than non-ACHD patients for a variety of cardiac and noncardiac diagnoses as well as for noncardiac surgery.^38^, ^39^, ^40^, ^41^ While these findings prompt suspicion that adults with CHD may have increased ICU mortality, evidence to date does not support this hypothesis. In a retrospective study of 138 nonelective ACHD ICU admissions in an adult tertiary care center with no dedicated cardiac ICU in the Netherlands, adults with CHD under age 40 had an ICU mortality rate of 4.8%, which is significantly lower than the 16.3% mortality rate observed in non-ACHD matched for age, gender, and admitting diagnosis.^42^ Similarly, in a multicenter analysis of 441 ACHD ICU admissions from a North American registry of dedicated adult cardiac ICUs, ICU mortality was 7.5% and in-hospital mortality was 12.7% for adults with CHD with no difference in ICU mortality when adjusted for age in comparison to adults with acquired heart disease.^7^ Thus, the higher morbidity associated with critical care for adults with CHD may not translate into greater ICU mortality across the entire population, with further work needed to identify subpopulations at the highest risk of mortality.

While the available evidence thus leaves some uncertainty regarding the comparative mortality of adults with CHD in the ICU, these studies provide valuable insights on predictors of mortality in this population. Admission diagnoses associated with the highest mortality in ACHD included cardiogenic shock, heart failure, and noncardiac medical problems (including liver or renal failure, hypoxic respiratory failure, and sepsis), and significant independent predictors of mortality included severe CHD and use of temporary MCS.^7^^,^^42^ Additionally, those adults with CHD who survive an ICU stay or inpatient hospitalization remain at increased risk of death postdischarge, with a long-term mortality of 12.5% over an 8-year median follow-up in one study, similar to that of adults with acquired heart disease.^42^ This natural history may be driven by accumulated cardiac and end-organ dysfunction that does not fully return to the preadmission baseline, leading to a cycle of recurrent admission with rapidly declining health and quality of life.

### Cardiac surgical ICU admissions

Among adults with CHD admitted to the ICU after cardiac surgery, morbidity and mortality are highly dependent on individual procedural and patient factors.^43^, ^44^, ^45^, ^46^ While overall in-hospital mortality for adults with CHD requiring surgical treatment ranges from 1% to 7%, certain subgroups are at higher risk, such as those with single ventricle physiology and those undergoing heart transplantation.^17^^,^^46^^,^^47^ Estimates for major complications after cardiac surgery range from 22% to 46%, with cardiac and respiratory complications being most common.^48^^,^^49^ Within a given subset of ACHD cardiac surgical patients, important predictors of adverse outcomes include extremes of body mass index, complex cardiac anatomy, hepatic dysfunction, three or more prior sternotomies, prolonged ventilatory wean, acute kidney injury requiring dialysis, and need for temporary MCS.^44^^,^^46^^,^^50^ In data comparing ACHD surgical and postsurgical care in adult vs pediatric centers, overall morbidity and mortality rates are similar.^51^, ^52^, ^53^ However, adults with CHD experienced significantly reduced mortality when surgery was performed by trained congenital cardiac surgeons.^53^^,^^54^

Though direct comparison of outcomes between ACHD and non-ACHD cardiac surgical patients is confounded by underlying differences in surgical procedures, available data suggest that adults with CHD have higher in-hospital mortality and higher postoperative complication rates.^55^^,^^56^ A National Inpatient Sample study comparing ACHD cardiac surgical patients to patients undergoing coronary artery bypass grafting revealed that adults with CHD had higher in-hospital mortality and increased neurologic, thromboembolic, and arrhythmic complications as well as increased rates of liver failure and sepsis.^55^

For adults with CHD undergoing cardiac surgery, the preponderance of evidence speaks to increased overall hospital costs and length of stay compared to non-ACHD cohorts.^55^^,^^56^ Data from 1764 ACHD admissions from the Pediatric Cardiac Critical Care Consortium registry identified that 8.8% of ACHD experienced an ICU length of stay ≥7 days, with predictors of longer stay including high complexity surgery, 3 or more prior sternotomies, and preoperative renal dysfunction or dialysis.^57^

Outcomes related specifically to the use of cardiac replacement therapies in ACHD vs non-ACHD merit specific consideration. Adults with CHD undergoing heart transplantation have a 1-year post-transplant mortality rate ranging from 10% to 20%, nearly double that of non-ACHD counterparts.^58^, ^59^, ^60^, ^61^ This early postoperative mortality is primarily attributable to issues that may arise in the cardiac surgical ICU such as graft failure, infection, and multisystem organ failure.^59^^,^^62^ Importantly, transplanted adults with CHD demonstrate a well-described “survival paradox,” in that those who survive to 1-year post-transplant have improved survival compared to patients receiving transplants for other indications.^59^^,^^63^ Similarly, adults with CHD undergoing durable ventricular assist device implantation have higher postoperative mortality compared to non-ACHD, with an increased rate of death predominantly due to operative and postoperative complications in the first 5 months after surgery.^64^ Those who survive this initial postsurgical period attain survival rates equal to non-ACHD patients, attesting to the critical nature of the postoperative ICU period as a determinant of long-term outcomes. Moreover, as ICUs become increasingly accustomed to the care of adults with CHD receiving temporary MCS for the indication of bridge-to-transplant,^65^ it is foreseeable that the use of MCS for nontransplant indications will rise in parallel.

## Key gaps in evidence and research priorities for care of ACHD patients in the ICU

Multiple key gaps in evidence are readily apparent. From an epidemiologic perspective, continued vigilance is critical to capture the changing demographics of the ACHD population. As medical care advances, this population is growing in total size, developing greater physiologic complexity,^66^ and advancing in age, with resultant increase in the typical comorbidities seen in older adults with acquired heart disease. Overall growth of the population may continue to put stress on an already constrained and overburdened ICU system. Finally, an aging ACHD population will accrue both typical sequelae of acquired heart disease as well as noncardiac illness that may require ICU care.^67^

There is an opportunity to leverage the growing number of accredited ACHD providers, nascent prospective registries, administrative databases, and machine learning to improve risk prediction and timely intervention to better ICU outcomes for adults with CHD. In recent years, several risk-prediction scores were developed and validated to predict outcomes for adults with CHD undergoing cardiac surgery, including the PErioperative ACHd (PEACH) Score and the ACHD-specific Society of Thoracic Surgeons risk score.^17^^,^^68^ Prospective studies are needed to determine if routine prospective assessment using these tools can be used to identify and mitigate perioperative risk to improve surgical outcomes. Furthermore, there has been minimal use of existing risk prediction models for nonsurgical admissions, such as the Society for Cardiovascular Angiography and Interventions Shock Classification, to identify risk a priori at ICU admission for ACHD patients and thereby tailor ICU therapies.^69^

Finally, there is an unmet need to determine which adults with CHD may benefit from invasive hemodynamic monitoring and what is the appropriate duration of indwelling catheters in those with shunt lesions. Certainly, there are specific anatomies (eg, shunt physiology, Fontan circulation, and multilevel left-sided outflow tract obstruction) in which invasive monitoring can be extremely informative. Evidence from the Cardiac Critical Care Trials Network suggests that ACHD patients are subject to increased use of pulmonary artery catheters and invasive hemodynamic monitoring compared to adults with acquired heart disease.^7^ However, it is not clear if this use is associated with improved outcomes, as has been shown in certain populations with advanced heart failure,^70^ or increased harm, as has been shown for patients following cardiac surgery.^71^

While there has been a gradual increase in the use of temporary and durable MCS over time,^65^^,^^72^ the field has yet to realize the full potential of these therapies in the setting of the myriad unique congenital anatomies and attendant physiology, with particular attention to single ventricle heart disease and those with a systemic RV. Support strategies using MCS may become an increasingly viable pathway to bridging to successful heart transplantation.

## Workforce and educational priorities for care of ACHD patients in the ICU

Over the last 2 decades, changes in ACHD care delivery systems have demonstrated improved outcomes and reduced mortality when these patients are cared for in ACHD centers of excellence with the availability of expert consultation by ACHD-trained providers.^53^^,^^54^^,^^73^^,^^74^ Early experience suggests that the development of collaborative ACHD ICU care models may similarly enhance outcomes, as a single-center quality improvement study demonstrated reduced in-hospital and long-term morbidity and mortality for postsurgical ACHD patients after the implementation of a dedicated ACHD ICU team.^75^

**Figure 4:**
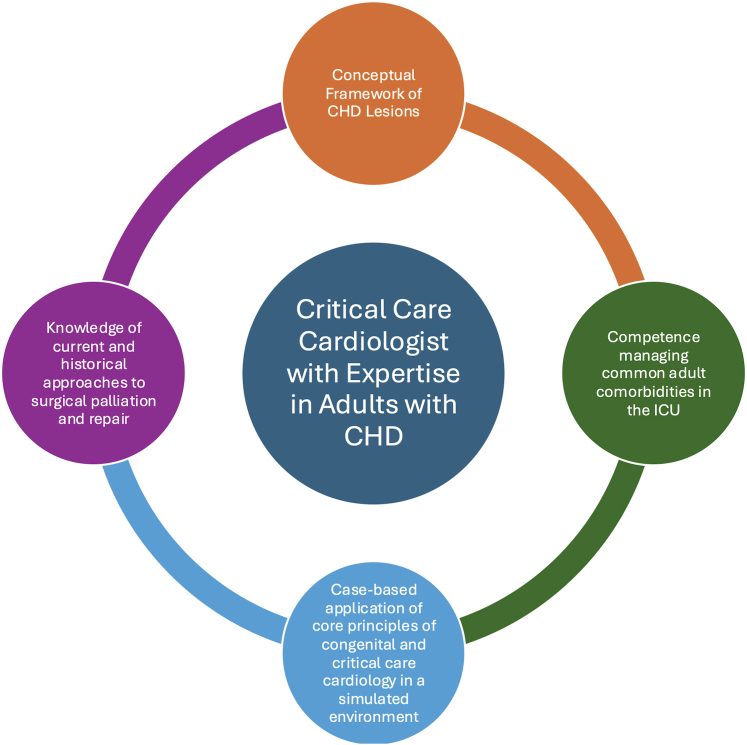
**Workforce Development in the Critical Care of Adults With CHD** Core educational priorities are proposed for developing a workforce of critical care cardiologists with expertise in caring for adults with congenital heart disease (CHD) in the intensive care unit (ICU).

There are currently a myriad of training pathways for physicians to find themselves caring for adults with CHD in the ICU. Yet, there is no standardized curriculum or competency-based training to ensure a “minimum necessary” knowledge to care for this unique and physiologically complex population. Critical care of this population requires an in-depth understanding of the complexities of congenital cardiology, breadth of repairs and palliative surgeries, grasp of the management of common adult noncardiac comorbidities, all while contextualizing care in a shared decision-making model with adults living with a lifelong condition. Such a curriculum would need to span across multiple disciplines that provide ICU care to adults with CHD and offers a significant opportunity for collaboration. For practitioners who wish to establish particular expertise, one could envision an educational framework that helps critical care cardiologists develop a foundational knowledge of congenital anatomy and physiology, key diagnostic findings, and interventional and surgical procedures unique to this population (Figure 4). Such a program could create a common educational framework for intensivists of all training backgrounds who care for adults with CHD in most ICU settings.

While it might be ideal for a single individual to be facile with all requirements needed to care for adults with CHD in the ICU, there are only a handful of intensivists in the United States who have completed formal ACHD training.^21^ As such, ICUs should focus on building highly functional teams that excel at both ACHD and ICU care. In the common scenario of lack of a dedicated ACHD-ICU provider, the consulting ACHD team, when available, should be an integral part of the daily rounds on the adults with CHD in the ICU. Additional support mechanisms can be developed to further improve care for critically ill adults with CHD. For example, in our program, we have developed a system to trigger automatic consultation of an additional team member when an unexpected clinical change is recognized, known as “red rules.” These mutually agreed upon “red rules” are few in number, easy to remember, and focus on patient safety in episodes of acute decompensation. For centers in the community without access to expert ACHD consultation, outreach efforts should be made to educate on which patients are highest risk and may be best served by referral to regional centers with ACHD expertise. Educational programs should also include advanced practice providers engaged in direct patient care, bedside nurses, nurse educators, as well as ancillary providers such as physical and occupational therapists whose contributions are integral to optimizing ICU outcomes for this population. Each of these stakeholders has unique knowledge, skillsets, licensing requirements, and experiences to bring to bear on the care of adults with CHD in the ICU. Setting aside the diversity in terms of care role and training background, it is imperative to recruit a diverse workforce, in terms of race, gender, ethnicity, and sociodemographic status.^76^^,^^77^

## Conclusions

While adults with CHD currently represent a small percentage of admissions to ICUs, the prevalence of adults with CHD in ICUs will continue to grow as this population ages and develops increasingly complex cardiac and noncardiac conditions. Considering the current heterogeneity in practice patterns of critical care for adults with CHD, there are opportunities to develop evidence-based, standardized clinical practice guidelines and care delivery models with the goal of improving outcomes for adults with CHD across all types of ICUs. As cardiac and critical care societies work together to define educational pathways, training requirements, and core competencies for critical care cardiologists, ACHD-focused components are likely to be valuable to develop an ICU workforce positioned to provide the best possible care for adults with CHD.

## Funding support and author disclosures

Dr Morrow is a member of the TIMI (Thrombolysis in Myocardial Infarction) Study Group, which has received institutional research grant support through Brigham and Women’s Hospital from 10.13039/100000046Abbott, 10.13039/100020297Abiomed, 10.13039/100002429Amgen, 10.13039/100020132Anthos Therapeutics, AstraZeneca, Bayer HealthCare Pharmaceuticals, Inc, Daiichi Sankyo, Eisai, 4TEEN4, 10.13039/100014130Intarcia, MedImmune, 10.13039/100004334Merck, 10.13039/100008272Novartis, 10.13039/100004319Pfizer, Quark Pharmaceuticals, 10.13039/100009857Regeneron Pharmaceuticals, Inc, Roche, Siemens Healthcare Diagnostics, Inc, and Zora Biosciences. Dr Morrow has received consulting fees from 10.13039/100001316Abbott Laboratories, 10.13039/100004334Merck & Co, 10.13039/100008272Novartis, 10.13039/100009857Regeneron, and 10.13039/100016545Roche Diagnostics. All other authors have reported that they have no relationships relevant to the contents of this paper to disclose.
